# Contraception Use in Adolescents and Young Adults with Congenital Heart Disease

**DOI:** 10.1007/s00246-025-03791-y

**Published:** 2025-03-03

**Authors:** Jaclyn Giafaglione, May Ling Mah, Lydia K. Wright

**Affiliations:** Division of Cardiology, Department of Pediatrics, The Heart Center, Nationwide Children’s Hospital, Ohio State University, 700 Children’s Drive, Columbus, OH 43205 USA

**Keywords:** Congenital heart disease (CHD), Single ventricle (SV), Contraception, Long-acting reversible contraception (LARC)

## Abstract

Women with congenital heart disease (CHD) are at elevated risk for morbidity and mortality during childbirth. Pediatric cardiologists are in a unique position to provide reproductive counseling to their patients with CHD. We evaluated contraception use in adolescents and young adults with CHD seen in a pediatric cardiology practice. Utilizing retrospective chart review, we evaluated all encounters for female patients aged 14 – 21 years with CHD between January 2017 and June 2023 at a tertiary care center. Female patients without CHD seen over the same period were included as a comparator group. Logistic regression was used to evaluate predictors of contraception use. There were 12, 368 visits included in our study. Of the 9924 visits in patients without CHD, 23% were on contraception, and of the 2444 visits in patients with CHD, 22% were on contraception. There was an increase in contraception use over time. Controlling for age at visit, later visit year was associated with contraception use (OR 1.09 [95% CI 1.04–10.9] per year). CHD was associated with lower likelihood of contraception use [OR 0.79 [95% CI 0.70 – 0.88]). Those with single ventricle (SV) CHD and complex CHD used long-acting reversible contraception more often (54% and 46% respectively) compared to those with simple or moderate CHD (17% and 16% respectively) or no CHD (18%). Patients with CHD are receiving contraception at a lower rate than those without CHD. Given the risks that women with CHD face with pregnancy, there should be a dedicated effort to increase the percentage of women with CHD on contraception.

## Introduction

Congenital heart disease (CHD) is the most commonly diagnosed congenital disorder in newborns worldwide. In the United States, CHD affects nearly 1% of births per year. In 2010, it was estimated that there were 2.4 million people living with CHD — 1.26 million of whom were women [1]. Women with CHD face significant physiologic challenges during pregnancy and delivery. Over time, we have seen that women with CHD, specifically those with complex lesions, are at high risk for morbidity and mortality during child birth [2]. Because of this, comprehensive counseling regarding contraception and risk of childbirth is paramount for young women with CHD. Despite this fact, there is an unmet need for contraception among adolescents and young adults with CHD.

The teen birth rate in the United States in 2019 was 16.7 per 1000, 82% of which were unplanned [3]. Among women with CHD, studies suggest 25% to 45% of pregnancies may be unintended [4]. In a multi-institutional study, approximately 77% of sexually active adolescents with CHD reported using contraception with intercourse; however, more than half were only using barrier contraceptive methods [5].

The American College of Obstetricians and Gynecologists (ACOG) and the American Heart Association (AHA) recommend contraceptive counseling for women of reproductive age who have CHD [6]. Specifically, ACOG recommends that “gynecologic care of adolescent girls and young women with cardiac conditions should occur in collaboration with the patient’s cardiologist [6].” Pediatric cardiologists are in a unique position to provide reproductive counseling to their adolescent patients with CHD given their understanding of the complex physiology and limitations that come with their underlying diagnosis. However, pediatric cardiologists have limited training on how to approach this conversation with their patients [7]. Therefore, we aimed to assess the use of contraception in female adolescent patients with CHD compared to their peers.

## Methods

Utilizing retrospective chart review, we evaluated all encounters for female patients aged 14 – 21 years with CHD between January 2017 and June 2023 at a tertiary care center. Female patients of similar age without CHD seen over the same period in the same clinic were included as a comparator group. Patients diagnosed with cardiomyopathies, arrhythmias or post orthotopic heart transplant were excluded. Included patients were categorized utilizing the Adult Congenital Heart Disease (ACHD) classification system into CHD of simple, moderate or severe complexity [8]. SV patients were separately analyzed. Baseline characteristics at the time of visit for each adolescent based on CHD severity were described using counts and percentages for categorical variables, and median and interquartile ranges for continuous variables. Characteristics were then compared between those visits in which the patient was on any form of contraception (study’s primary outcome) and those in which the patient was not on any form of contraception. Student’s T-tests were used to compare mean and standard deviation (SD) for normally distributed continuous variables, Wilcoxon rank-sum tests were used to compare median and interquartile ranges (IQR) for non-normally distributed continuous variables, and chi-square tests were used to compare frequencies and percent for categorical variables. Logistic regression models were constructed to evaluate the likelihood of our primary outcome, with potential confounders selected a priori, including age, year of visit, and patient’s race. Trend in contraception use over time was evaluated for each diagnosis group, as was type of contraception prescribed. Long-acting reversible contraception–use, defined as the presence of intrauterine device (IUD) or implant, was also evaluated for each group.

## Results

During the study period, a total of 12,368 visits from 7819 unique patients were included. Of those, 2444 (19.8%) of visits were for patients without CHD and 9924 (80.2%) were for patients with CHD. Demographic characteristics stratified by the presence and type of CHD are shown in Table 1. Age at visit was similar across all patient groups, with a median age of 16 years. A total of 698 (6%) visits were in adolescents with simple CHD, 1357 (11%) with moderate CHD, 243 (2%) with Complex CHD, and 146 (1%) with SV CHD. The number of visits per patient across the study period increased with increasing complexity, with those without CHD having a median of one visit (IQR 1 – 3), complex CHD having a median of 6 visits (IQR 3 – 10), and those with SV having a median of 5 visits (4 – 8). The majority of patients in all groups was white with commercial health insurance. There were more visits per patient in individuals with moderate, complex and SV heart disease as compared to visits in those with simple or no CHD.

**Table 1:** - Demographic information of study participants categorized by type of CHD

Characteristic	No CHD, N = 9924^*1*^	Simple, N = 698^*1*^	Moderate, N = 1357^*1*^	Complex, N = 243^*1*^	SV, N = 146^*1*^
Age at visit	16 (15, 17)	16 (15, 17)	16 (15, 18)	16 (15, 18)	16 (14, 17)
Race					
White	7547 (76%)	539 (77%)	1133 (83%)	167 (69%)	112 (77%)
Black	1201 (12%)	71 (10%)	115 (8.5%)	43 (18%)	22 (15%)
Asian	169 (1.7%)	39 (5.6%)	37 (2.7%)	8 (3.3%)	9 (6.2%)
Other	711 (7.2%)	42 (6.0%)	61 (4.5%)	20 (8.2%)	2 (1.4%)
Unknown	296 (3.0%)	7 (1.0%)	11 (0.8%)	5 (2.1%)	1 (0.7%)
Insurance					
Commercial	6261 (63%)	397 (57%)	890 (66%)	135 (56%)	84 (58%)
Medicaid	3165 (32%)	249 (36%)	361 (27%)	86 (35%)	46 (32%)
Other	305 (3.1%)	29 (4.2%)	67 (4.9%)	12 (4.9%)	11 (7.5%)
Unknown	193 (1.9%)	23 (3.3%)	39 (2.9%)	10 (4.1%)	5 (3.4%)
Visits per patient	1 (1, 3)	2 (1, 4)	4 (2, 6)	6 (3, 10)	5 (4, 8)

^*1*^Median (IQR); n (%)

**Fig. 1:**
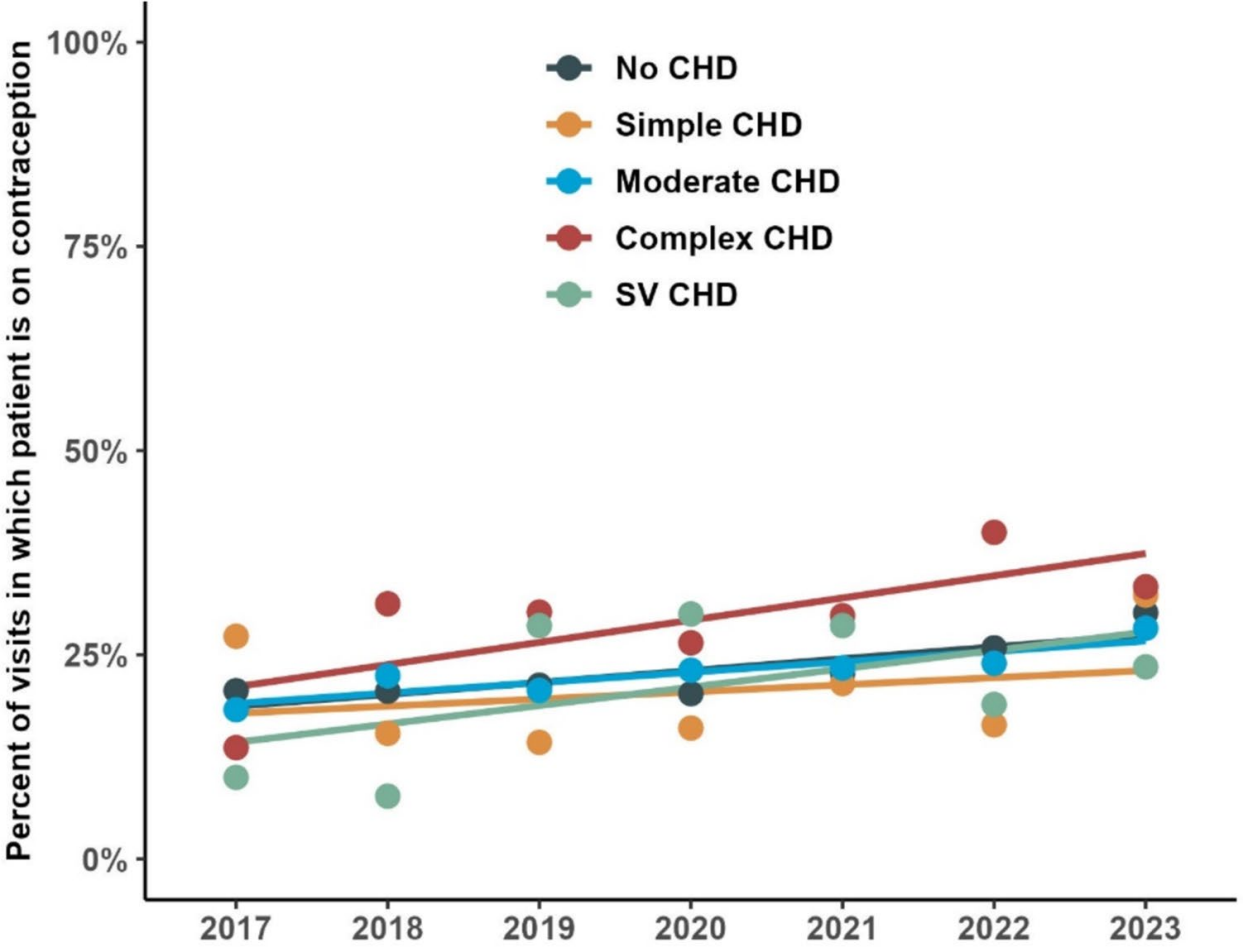
Rate of contraception use per year for each CHD category

Significant differences regarding patient’s characteristics in visits in which patients were on contraception compared to not receiving contraception were observed (Table 2). Those on contraception were significantly older (median age 17 years vs 16 years, p < 0.001) and more likely to be white (p < 0.001). There was also a difference in diagnosis; specifically the highest rates of contraception were observed in those with complex CHD (30%), whereas patients with simple CHD were the least (19%). There was a steady increase in percentage of patients on contraception by year, starting in 2021. Figure 1 demonstrates this change over time based on type of CHD; there was a trend toward increasing contraception use among all groups.

**Table 2:** Characteristics stratified by contraception status at visit

Characteristic	Not on contraception, N = 9529^*1*^	On contraception, N = 2839^*1*^	p-value^*2*^
Age at visit	16 (14, 17)	17 (16, 18)	< 0.001
Race			< 0.001
White	7140 (75%)	2358 (83%)	
Black	1230 (13%)	222 (7.8%)	
Asian	230 (2.4%)	32 (1.1%)	
Other	682 (7.2%)	154 (5.4%)	
Unknown	247 (2.6%)	73 (2.6%)	
Insurance			0.015
Commercial	5949 (62%)	1818 (64%)	
Medicaid	3051 (32%)	856 (30%)	
Other	309 (3.2%)	115 (4.1%)	
Unknown	220 (2.3%)	50 (1.8%)	
CHD category			0.019
No CHD	7635 (80%)	2289 (81%)	
Simple	562 (5.9%)	136 (4.8%)	
Moderate	1049 (11%)	308 (11%)	
Complex	170 (1.8%)	73 (2.6%)	
SV	113 (1.2%)	33 (1.2%)	
Visit year			< 0.001
2017	1159 (12%)	299 (11%)	
2018	1240 (13%)	322 (11%)	
2019	1434 (15%)	384 (14%)	
2020	1366 (14%)	353 (12%)	
2021	1691 (18%)	504 (18%)	
2022	1689 (18%)	569 (20%)	
2023	950 (10.0%)	408 (14%)	

^*1*^Median (IQR); n (%)

^*2*^Wilcoxon rank–sum test; Pearson's chi-squared test

Multivariable logistic regression model of likelihood of being on contraception at visit is shown in Table 3. When compared to patients without CHD, those with CHD were significantly less likely to be prescribed contraception [OR 0.79 (95% CI 0.70 to 0.88)]. This result was adjusted for differences in age and year of visit, as both increased age and later year of visit were associated with increased odds of contraception use (p < 0.001 for both). Of note, patients’ race was also associated with contraception use, with all non-white adolescents being less likely to be on contraception compared to their white peers of similar age, diagnosis, and year of visit.

**Table 3:** Multivariable logistic regression model of likelihood of being on contraception at visit

Characteristic	OR^*1*^	95% CI^*1*^	p-value
Diagnosis			
No CHD	—	—	
Simple	0.70	0.57, 0.85	< 0.001
Moderate	0.76	0.66, 0.88	< 0.001
Complex	1.18	0.88, 1.59	0.3
SV	0.82	0.53, 1.22	0.3
Age at Visit	1.41	1.38, 1.45	< 0.001
Visit year	1.07	1.04, 1.09	< 0.001
Race			
White	—	—	
Black	0.51	0.44, 0.60	< 0.001
Asian	0.39	0.26, 0.56	< 0.001
Other	0.71	0.58, 0.85	< 0.001
Unknown	0.99	0.75, 1.29	> 0.9

^*1*^OR odds ratio, *CI* confidence interval

**Fig. 2:**
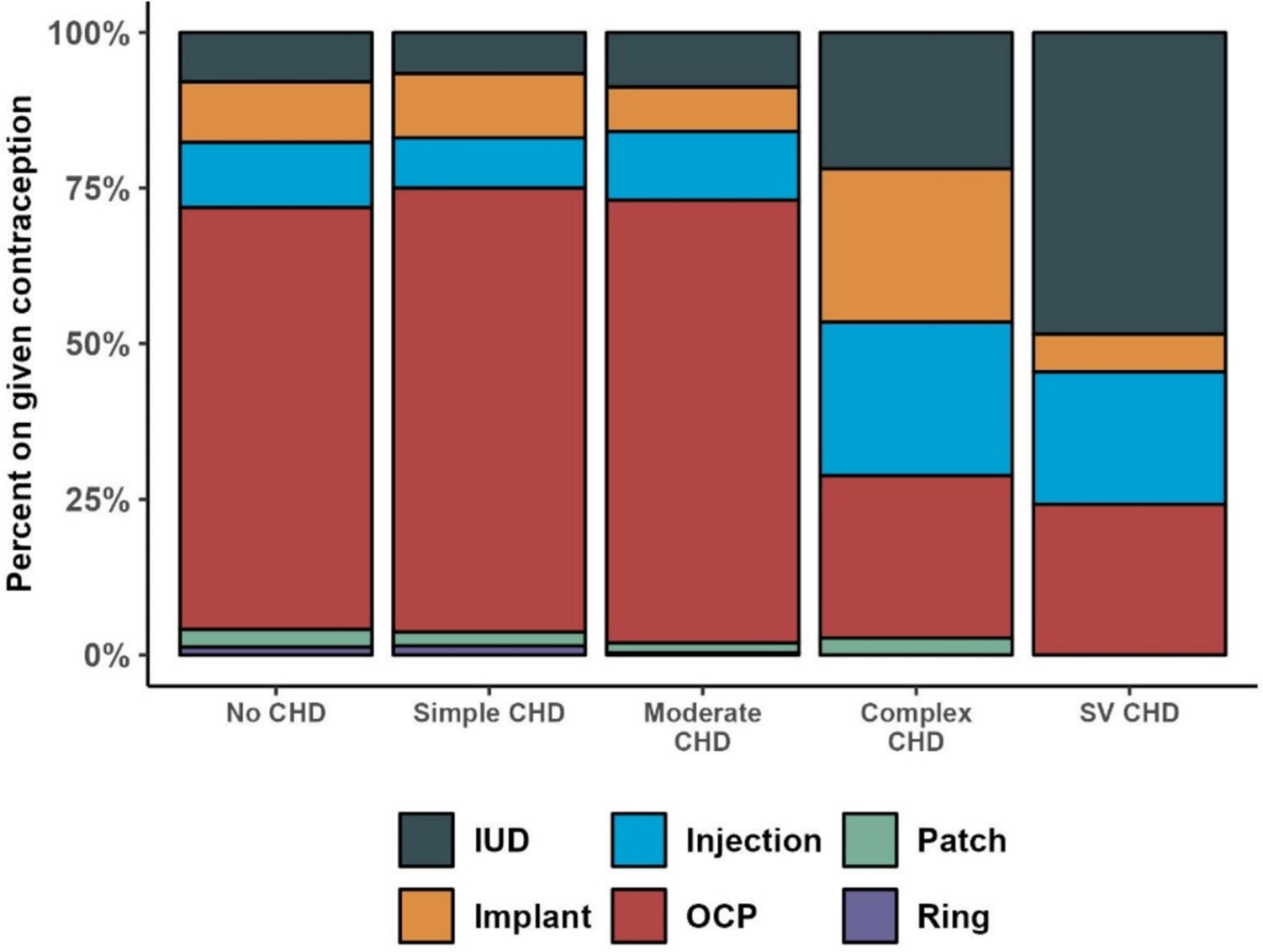
Type of contraception by CHD category for those on contraception. **IUD Intrauterine Device, *OCP* oral contraceptive pill

Type of contraception by underlying CHD is shown in Fig. 2. Oral contraceptive pills were most used in those with no CHD and those with less complex CHD. The majority of patients with SV CHD who were on contraception (55%) were on some form of long-acting reversible contraception (LARC), i.e., intrauterine devices (IUDs) or implants as compared to 18% in those without CHD 17% in those with simple CHD, 16% in those with moderate CHD and 46% in those with complex CHD (p =  < 0.001).

## Discussion

Women with CHD face significant challenges during pregnancy compared to women without heart disease. Despite this fact, preconception and contraceptive counseling has not been a priority for adolescent female with CHD. Our data demonstrate that adolescents with a diagnosis of CHD were significantly less likely to be on contraception than similar aged peers without CHD seen in cardiology clinic. Some have postulated that adolescents with CHD are delayed in maturity as compared to their peers and therefore are not as sexually active as the general population. Fry et al. did demonstrate that, compared to the general population, a lower percentage of adolescents were engaging in sexual intercourse [5]. However, they also demonstrated that those being sexually active were primarily using barrier contraception as birth control. When this strategy is used correctly, it results in 13–21 pregnancies per 100 women-years [5, 9]. Given the significant morbidity and mortality of patients with CHD, especially in those with complex CHD and SV physiology, a more reliable and safe form of contraception should be discussed with these patients.

Although there are still more patients with CHD not on contraception at our institution, the trend is encouraging, as there has been an increase in contraception use over time (Fig. 1). Furthermore, those on contraception having complex CHD and SV physiology (who are at the highest risk of adverse pregnancy outcomes) are utilizing LARC methods compared to less reliable methods as seen in Fig. 2. The goal should be to continue to improve contraceptive counseling and access for patients with CHD. Cardiologists, cardiology fellows and advanced practitioners have reported low levels of confidence when discussing pregnancy complications and contraceptive options for their patients with CHD [10]. Education for these providers is paramount to provide better counseling for these patients.

This study also demonstrated important racial disparities regarding contraception use. All non-white patients had significantly lower odds of using contraception, highlighting the necessity for improving inequalities. This emphasizes the importance for culturally sensitive education to increase contraceptive counseling and access for all patient populations.

We are just touching the surface surrounding this important topic, given that the study is a single–institution, retrospective in nature. Additional limitations include lack of granularity in diagnostic codes. Categorization of CHD was based on visit diagnoses, and hence a patient could have been categorized as simple complexity whereas their CHD lesion may be more complex. There were also certain diagnoses like “mitral valve prolapse” that were not listed under the ACHD categories, so the authors defined these conditions based on their personal knowledge and experience.

In conclusion, female patients with CHD require improved counseling and access to contraception given the substantial risk they face during pregnancy and delivery. Cardiologists are in a unique position to provide this counseling based on their understanding of the underlying anatomy and physiology that many of these patients live with.

## Data Availability

The data that support the findings of this study are not openly available due to reasons of sensitivity. Data are, however, available from the authors upon reasonable request and with permission from the Institutional Review Board at Nationwide Children's Hospital.
